# Beyond Pairwise Interactions: How Other Species Regulate Competition Between Two Plants?

**DOI:** 10.3390/plants14132018

**Published:** 2025-07-01

**Authors:** Wang-Xin Cheng, Wei Xue, Jie-Jie Jiao, Hao-Ming Yuan, Lin-Xuan He, Xiao-Mei Zhang, Tao Xu, Fei-Hai Yu

**Affiliations:** 1 College of Life Science and Medicine, Zhejiang Sci-Tech University, Hangzhou 310018, China; wangxincheng1121@163.com (W.-X.C.); xutao@zstu.edu.cn (T.X.); 2 Institute of Wetland Ecology & Clone Ecology/Zhejiang Provincial Key Laboratory of Evolutionary Ecology and Conservation, Taizhou University, Taizhou 318000, China; x_wei1988@163.com (W.X.); haomingyuan629@163.com (H.-M.Y.); lamhimho@163.com (L.-X.H.); xiaomeiz2022@163.com (X.-M.Z.); 3 Zhejiang Academy of Forestry, Hangzhou 310023, China; jjj029302@hotmail.com

**Keywords:** competitive effect, interspecific interactions, multi-species interactions, neighbor effect, plant–plant interactions, species coexistence

## Abstract

A plant species in a community often grows with some other plant species. While many studies have assessed interspecific interactions between two target plant species, few have considered the impacts of the other plant species (e.g., the third, fourth, and fifth plant species) on these interactions. To assess the impacts, we grew one seedling of each of the five herbaceous plant species that are common in China (*Cynodon dactylon*, *Plantago asiatica*, *Taraxacum mongolicum*, *Nepeta cataria*, and *Leonurus japonicus*) alone (no competition) or with one seedling of one, two, three, or four of the other species. The presence of a neighbor plant generally reduced the growth of the target species, suggesting that the interspecific relationships were mostly competitive. The presence of other neighbor species (the third, fourth, and fifth species) could alter the interspecific interactions between two target species, but such effects varied depending on both the identity of the target species and the identity of the other species. Additionally, the effects of the third species depended little on the presence of the fourth and fifth species. We conclude that interspecific interactions between two plant species are commonly regulated by the presence of other species, facilitating species coexistence. However, our findings do not support the idea that the impacts of the fourth and fifth species on interactions among three plant species are common. This study highlights the complex interactions among multiple plant species within a community and also the importance of including these high-order interactions when modelling community dynamics and species coexistence.

## 1. Introduction

In natural ecosystems, species are rarely found in isolation; they typically grow and interact with a variety of other species [1,2]. Interactions among plant species are important determinants of species coexistence and community structure, and ecosystem function [3,4,5]. Competition is one of the most common plant–plant interactions and plays a key role in natural ecosystems, influencing species survival and growth, resource acquisition, population dynamics, and ecosystem stability [6,7,8]. Understanding factors regulating plant competition is important for developing predictive models of species coexistence and community dynamics [9,10,11].

Competitive interactions between two target plant species can be regulated by various environmental factors, including temperature [12,13,14], CO_2_ concentration [15], O_3_ concentration [16], water [17,18], light [19], nutrients [20,21], salinity [22], heavy metal contamination [23], and individual density [24]. For example, in a maize–soybean cropping system, maize typically outcompetes soybean; however, the addition of nitrogen and phosphorus fertilizers can reduce this competitive advantage [25]. Invasive species often outcompete native species, reducing native species’ richness and abundance, but such a competitive effect can be modified by light, soil water, and nutrients [26,27,28].

As a plant species often coexists with more than one plant species, indirect interactions between two plant species can also be essential for species coexistence and biodiversity maintenance [29]. One example of this is that competitive interactions between two target plant species can be modified by organisms from other tropic levels [30]. For instance, *Solanum altissima* and *Solidago carolinense* can coexist when insect herbivores are not present, but *S. altissima* may outcompete *S. carolinense* when the herbivores are present [31]. Also, compared to the absence of arbuscular mycorrhizal fungi, the presence of the fungi can increase the competitive ability of *Vicia faba* on *Hordeum vulgare* by improving the nitrogen uptake of *V. faba* [32]. Moreover, endophyte-infected plants show a higher competitive ability than those that are not infected [33].

The introduction of new plant species into a community can further complicate competitive interactions and, thus, coexistence between two target species [34,35]. In a recent study, the duckweed *Spirodela Polyrhiza* was found to suppress another duckweed, *Lemna minor*, when *Pistia stratiotes* was not present, but such a suppressing effect diminished when *P. stratiotes* was included [36]. Studies have also shown that both the species richness of a plant community and genotypic richness of a clonal plant population could alter competitive interactions between two target plant species [37,38]. However, as the number of other plant species increases, interspecific interactions between two target plant species may become more complex. So far, however, it is still unknown as to whether the impact of the other plant species (e.g., the third plant species) on competitive interactions between two target plant species is common and whether the impact varies depending on the presence of more additional plant species (e.g., the fourth and the fifth plant species).

To assess the impacts of other plant species on competitive interactions between two target plant species, we grew one seedling of each of the five herbaceous plant species that are common in China (*Cynodon dactylon*, *Plantago asiatica*, *Taraxacum mongolicum*, *Nepeta cataria*, and *Leonurus japonicus*) alone (no competition) or with one seedling of one, two, three, or four of the other species. Specifically, we address (1) how common the impact of the third plant species is on competition between two target plant species, (2) whether the impact of the third plant species varies depending on the identity of the target species and the identity of the third species, and (3) whether the impact of the third plant species varies depending on the presence of the fourth and the fifth species.

## 2. Results

**Figure 1:**
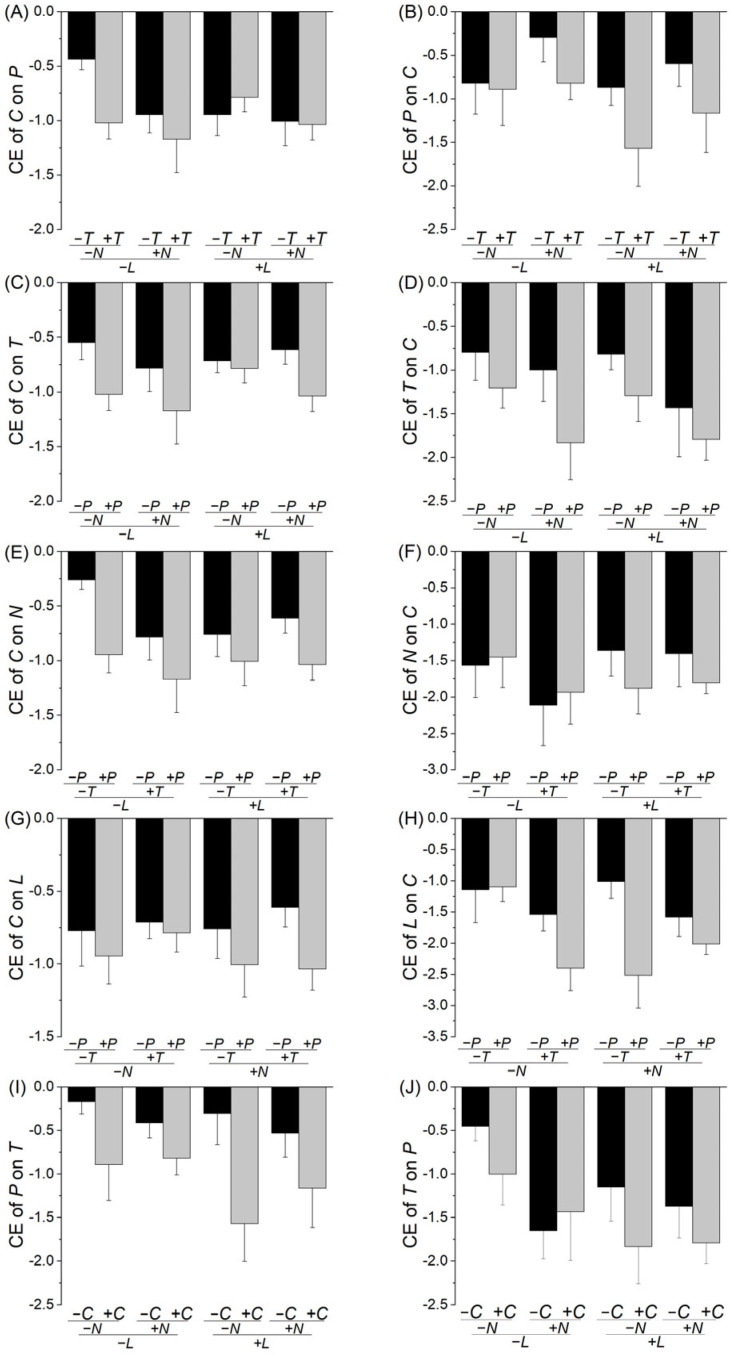
Effects of the presence of other species on the competitive effect of (**A**) *C* on *P*, (**B**) *P* on *C* (**C**) *C* on *T*, (**D**) *T* on *C*, (**E**) *C* on *N*, (**F**) *N* on *C*, (**G**) *C* on *L*, (**H**) *L* on *C*, (**I**) *P* on *T*, and (**J**) *T* on *P*. Bars and vertical lines are means ± SE. *C*, *P*, *T*, *N*, and *L* represent *Cynodon dactylon*, *Plantago asiatica*, *Taraxacum mongolicum*, *Nepeta cataria*, and *Leonurus japonicus*, respectively.

**Figure 2:**
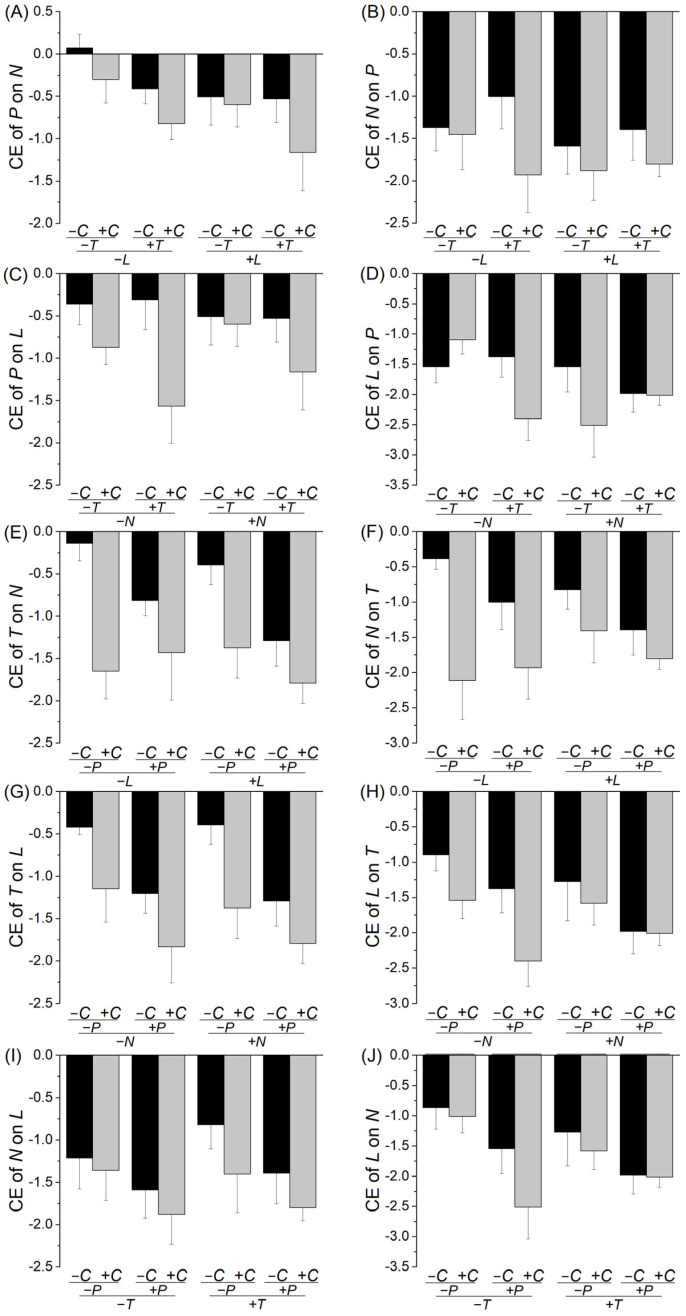
Effects of the presence of other species on the competitive effect of (**A**) *P* on *N*, (**B**) *N* on *P* (**C**) *P* on *L*, (**D**) *L* on *P*, (**E**) *T* on *N*, (**F**) *N* on *T*, (**G**) *T* on *L*, (**H**) *L* on *T*, (**I**) *N* on *L*, and (**J**) *L* on *N*. Bars and vertical lines are means ± SE. *C*, *P*, *T*, *N*, and *L* represent *Cynodon dactylon*, *Plantago asiatica*, *Taraxacum mongolicum*, *Nepeta cataria*, and *Leonurus japonicus*, respectively.

The presence of neighbor plants generally reduced the growth of the target species, as shown by the negative value of the log response ratio, suggesting that the interspecific relationships were mostly competitive (Figure 1 and Figure 2). The presence of the third species could alter the interspecific interactions between two target species (Table 1, Figure 1 and Figure 2). Specifically, the presence of *P. asiatica* aggravated (*p* < 0.05) or tended to aggravate (0.05 < *p* < 0.1) the competitive effect of *C. dactylon* on *T. mongolicum* (Table 1C, Figure 1C); *T. mongolicum* on *C. dactylon* (Table 1D, Figure 1D), *N. cataria* (Table 1O, Figure 2E) and *L. japonicus* (Table 1Q, Figure 2G); *C. dactylon* on *N. cataria* (Table 1E, Figure 1E) and *L. japonicus* (Table 1G, Figure 1G); *L. japonicus* on *C. dactylon* (Table 1H, Figure 1H), *T. mongolicum* (Table 1R, Figure 2H) and *N. cataria* (Table 1T, Figure 2J); and *N. cataria* on *L. japonicus* (Table 1S, Figure 2I). Similarly, the presence of *C. dactylon* aggravated (*p* < 0.05) or tended to aggravate (0.05 < *p* < 0.1) the competitive effect of *P. asiatica* on *T. mongolicum* (Table 1I, Figure 1I), *N. cataria* (Table 1K, Figure 2A), and *L. japonicus* (Table 1M, Figure 2C); *N. cataria* on *P. asiatica* (Table 1L, Figure 2B) and *T. mongolicum* (Table 1P, Figure 2F); *T. mongolicum* on *N. cataria* (Table 1O, Figure 2E) and *L. japonicus* (Table 1Q, Figure 2G); and *L. japonicus* on *T. mongolicum* (Table 1R, Figure 2H). However, the presence of *N. cataria* only tended to aggravate (0.05 < *p* < 0.1) the competitive effect of *C. dactylon* on *P. asiatica* (Table 1A, Figure 1A), and *T. mongolicum* on *P. asiatica* (Table 1J, Figure 1J); the presence of *L. japonicus* only aggravated (*p* < 0.05) the competitive effect of *T. mongolicum* on *C. dactylon* (Table 1D, Figure 1D); and the presence of *T. mongolicum* tended to aggravate (0.05 < *p* < 0.1) the competitive effect of *P. asiatica* on *C. dactylon* (Table 1B, Figure 1B) and *N. cataria* (Table 1K, Figure 2A), and *L. japonicus* on *C. dactylon* (Table 1H, Figure 1H).

We found a significant interaction effect of *C. dactylon* × *T. mongolicum* × *N. cataria* on the competitive effect of *L. japonicus* on *P. asiatica* (Table 1N): without *N. cataria*, the presence of *C. dactylon* weakened the competitive effect of *L. japonicus* on *P. asiatica* in the absence of *T. mongolicum*, but strengthened it in the presence of *T. mongolicum*; with *N. cataria*, however, the presence of *C. dactylon* aggravated the competitive effect of *L. japonicus* on *P. asiatica* in the absence of *T. mongolicum*, but had no impact in the presence of *T. mongolicum* (Figure 2D). There was also a marginally significant effect of *P. asiatica* × *T. mongolicum* × *N. cataria* on the competitive effect of species *L. japonicus* on *C. dactylon* (Table 1H): without *N. cataria*, the presence of *P. asiatica* had no impact on the competitive effect of *L. japonicus* on *C. dactylon* in the absence of *T. mongolicum*, but aggravated it in the presence of *T. mongolicum*; with *N. cataria*, the presence of *P. asiatica* aggravated the competitive effect of *L. japonicus* on *C. dactylon*, but such an impact was much weaker in the presence than in the absence of *T. mongolicum* (Figure 1H). However, we found no other significant two- or three-way interaction effects (Table 1).

## 3. Discussion

While it is well known that interspecific interactions between two plant species can be regulated by many biotic and abiotic factors [15,16,19,22,23], including the presence of species from other trophic levels [31,32] and the presence of other plant species [39,40], relatively few studies have explored how the presence of multiple additional plant species modifies interspecific plant–plant interactions [37]. Understanding these multi-species effects is crucial for predicting species coexistence and community stability in natural ecosystems. Our results suggest that it may be a common phenomenon that the presence of one additional plant species in a community can alter the competition between two target plant species, but it may be uncommon that this impact depends also on the presence of more plant species (i.e., a fourth and/or fifth plant species). These findings highlight the complexity of multi-species interactions within a plant community.

While the impact of the third plant species on competition was found to be relatively common, it varied depending on the identity of the target species. For instance, the presence of *Plantago asiatica* aggravated the competitive effect of *Cynodon dactylon* on *Taraxacum mongolicum* (Table 1C, Figure 1C), *T. mongolicum* on *C. dactylon* (Table 1D, Figure 1D), and *C. dactylon* on *Nepeta cataria* (Table 1E, Figure 1E), but did not alter the competitive effect of *N. cataria* on *C. dactylon* (Table 1F, Figure 1F). This variation may be due to the fact that different target plant species may differ greatly in factors such as their competitive abilities, resource utilization strategies, and potential allelopathic effects [41,42]. For example, *C. dactylon* uses a strategy of rapid stolon expansion and its root system allows it to rapidly occupy limitedly soil space and acquire soil resources [43], but *N. cataria* is capable of releasing allelopathic compounds that affect the growth of neighboring plants, altering the competitive balance [44]. Consequently, the impact of the third species on the competitive effect differed among target species, reflecting the complexity of interspecific relationships [45,46].

Additionally, the impact of the third plant species on plant–plant competition varied with the identity of the third plant species. For example, the competitive effect of *C. dactylon* on *T. mongolicum* was not influenced by the presence of *N. cataria*, *Leonurus. japonicus*, *N. cataria*, or *L. japonicus*, but it was aggravated by the presence of *P. asiatica* (Table 1C, Figure 1C). The rosette growth form of *P. asiatica* enables it to quickly occupy aboveground space and intercept light at low canopy levels, which was particularly influential during the early stage of community in this experiment. This trait likely reduced light availability for neighboring seedlings, thereby modifying the growth performance and interspecific interactions of co-occurring species. Again, this context-dependent impact of the third species is likely due to the differences in, for example, competitive abilities and resource acquisition strategies among the third plant species involved [41,42]. These results suggest that different third species may play functionally distinct roles in modifying competition, either amplifying or mitigating the strength of interactions depending on their traits. These findings align with those of previous studies that emphasize the role of plant functional traits (e.g., root [47] or leaf area [48]) in shaping competitive dynamics. For instance, plant size asymmetry has been shown to influence competition strength, with larger individuals often exerting stronger suppressive effects on smaller competitors [36]. Similarly, species with extensive root systems and high resource acquisition rates tend to increase competition intensity, whereas species with complementary resource-use strategies reduce direct competition [49,50]. This could explain why some third species in our study intensified competition, while others had little or no effect. Additionally, indirect interactions mediated by belowground processes, such as root exudates influencing microbial activity, may have further contributed to these species–specific differences in competitive effects [51].

We observed that the impact of *P. asiatica* on competition between *L. japonicus* and *C. dactylon* and the impact of *C. dactylon* on competition between *L. japonicus* and *P. asiatica* varied depending on the presence of both *T. mongolicum* and *N. cataria* (Table 1H,N, Figure 1H and Figure 2D). These findings suggest that the presence of a fourth and/or a fifth plant species can influence the impact of the third plant species on plant–plant interactions. However, we found no other significant two- or three-way interaction effects (Table 1), suggesting that these impacts are not so common. One explanation is that increasing species richness may directly reduce the strength of individual pairwise interactions due to resource partitioning or niche differentiation, leading to a more diffuse interaction network [52,53,54,55]. Another explanation lies in the stabilization of interaction networks [56]. Previous studies have shown that competitive rings tend to be more stable when the number of species involved is odd rather than even [57], suggesting that community-level patterns of interaction may be sensitive to species number and composition [58,59]. This complexity highlights the non-additive nature of species interactions, where the presence of more species does not simply accumulate effects but reshapes the entire interaction web.

One caveat is that the current experimental design (additive design) does not allow us to exclude the potential cofounding effect of changing density [60,61], and the lack of morphological, physiological. and allocational measurements precludes the possibility to explore the underlying mechanisms. Therefore, a novel experimental design and morphological, physiological, and allocational measurements are required to better understand the complex multi-species interactions in a plant community. The experiment for lasted more than three months in the greenhouse, which is more than the period of most potting experiments. Also, the pots used were small (10 cm × 10 cm × 10 cm) and plant roots and the above-ground parts had filled the pot. Thus, the experiment duration was long enough to address our questions.

## 4. Materials and Methods

### 4.1. Plant Species

To address the questions, we chose five plant species, i.e., *Cynodon dactylon* (L.) Persoon (Poaceae), *Plantago asiatica* L. (Plantaginaceae), *Taraxacum mongolicum* Hand.-Mazz. (Asteraceae), *Nepeta cataria* L. (Lamiaceae), and *Leonurus japonicus* Houtt. (Lamiaceae) (Table 2; Flora of China, www.iplant.cn (accessed on 30 May 2023)). They were chosen because they are all herbaceous plant species that are common in China and can co-occur in, for example, hillside grasslands, abandoned fields, and roadsides in eastern China where the experiment was conducted. *C*. *dactylon* is cable of clonal growth by rooting at the nodes of its creeping stems, while all of the other four species are non-clonal plants. *C. dactylon*, *T. mongolicum*, and *N. cataria* are perennial, *P*. *asiatica* is biennial or perennial, while *L. japonicus* is annual or biennial.

Seeds of these species were purchased online from the Ferryman’s Seed Superstore (Zhoukou, Henan Province, China). In June 2023, we sowed the seeds of the five species in trays (54 cm long × 28 cm wide × 5 cm deep) filled with peat and placed the trays in a greenhouse at Taizhou University in Taizhou, Zhejiang Province, China. After one month of germination and growth, for each species, more than 96 seedlings of a similar size were selected and used for the experiment described below.

### 4.2. Experimental Design

The competition experiment used an additive design. One seedling of each of the five species was grown alone (no competition) in a pot or with one seedling of one, two, three or four of the other species in a pot, resulting in a total of 31 treatments (species combinations; Table 3). Each treatment was replicated six times, resulting in a total of 186 pots.

On 24 July 2023, we transplanted the seedlings into square pots (10 cm × 10 cm × 10 cm) filled with a 2:1:1 (*v*:*v*:*v*) mixture of peat, river sand, and a local soil, and covered the pots with shading nets to facilitate initial seedling establishment. The local soil was collected from the mountainous area of Taizhou and contained 0.62 ± 0.17 (mean ± SE) g kg^−1^ total nitrogen and 0.13 ± 0.03 g kg^−1^ total phosphorus. During the first two weeks, the survival status of each seedling was monitored daily and dead seedlings were replaced immediately. The shading net was then removed. During the experiment, we watered the plants once the soil surface of most pots dried out and supplied 50 mL of a 10% Hoagland nutrient solution to each pot once a month [62].

### 4.3. Harvest and Measurement

We harvested the aboveground parts of each species in each pot on 2 November 2023, after more than three months of growth. All plant materials were oven-dried at 70 °C for at least 48 h and weighed to obtain the biomass.

### 4.4. Data Analysis

To directly quantify the competitive effect, we calculated the log response ratio as follows: LogRR_ij_ = ln (Bij/Bi), where LogRR_ij_ is the log response ratio of species j on species i, Bij represents the aboveground biomass of species i in the presence of species j for each replicate, and Bi represents the mean aboveground biomass of target species i when grown alone (no competition) across the replicates. A three-way ANOVA was performed to analyze the effect of the presence each of the other three species (the 3rd species) and their two-way and three-way interactions on the competitive effect (LogRR) of the two target species. A significant two-way interaction effect indicates that the effect of the 3rd species on the competition between two target species depends on the presence of the 4th species, and a significant three-way interaction effect means that the effect of the 3rd species on the competition between two target species depends also on the presence of the 5th species. All analyses were carried out using SPSS Statistics 26.0 software (IBM Corp., Armonk, NY, USA).

## 5. Conclusions

We conclude that interspecific interactions between two plant species in a multi-species community may be commonly regulated by the presence of other species. However, it seems uncommon that the impact of the third plant species on competition is regulated by the presence of the fourth and/or the fifth plant species. This study highlights the complex interactions among multiple plant species within a community and also the importance of including these high-order interactions when modelling community dynamics and species coexistence.

## Figures and Tables

**Table 1:** Effects of the presence of other species (the third, fourth, and fifth species) on the competitive effect of one target species on the other. *C*, *P*, *T*, *N*, and *L* represent *Cynodon dactylon*, *Plantago asiatica*, *Taraxacum mongolicum*, *Nepeta cataria*, and *Leonurus japonicus*, respectively. Values are in bold when *p* < 0.05 and in italics when 0.05 < *p* < 0.1.

Effect	*F*	*p*	Effect	*F*	*p*
(A) Competitive effect of species *C* on *P*			(B) Competitive effect of species *P* on *C*		
*T*	1.655	0.206	*T*	3.773	*0.059*
*N*	3.394	*0.073*	*N*	1.781	0.190
*L*	0.141	0.709	*L*	2.036	0.161
*T* × *N*	0.103	0.750	*T* × *N*	0.114	0.738
*T* × *L*	3.191	*0.082*	*T* × *L*	0.489	0.488
*N* × *L*	0.437	0.512	*N* × *L*	0.008	0.928
*T* × *N* × *L*	1.075	0.306	*T* × *N* × *L*	0.370	0.547
(C) Competitive effect of species *C* on *T*			(D) Competitive effect of species *T* on *C*		
*P*	7.243	**0.010**	*P*	3.956	*0.054*
*N*	1.130	0.294	*N*	0.262	0.612
*L*	0.554	0.461	*L*	4.477	**0.041**
*P* × *N*	0.279	0.600	*P* × *N*	0.085	0.772
*P* × *L*	0.536	0.468	*P* × *L*	0.098	0.756
*P* × *L*	0.230	0.634	*P* × *L*	0.172	0.680
*P* × *N* × *L*	0.761	0.388	*P* × *N* × *L*	0.305	0.584
(E) Competitive effect of species *C* on *N*			(F) Competitive effect of species *N* on *C*		
*P*	10.012	**0.003**	*P*	0.287	0.595
*T*	1.307	0.260	*T*	0.730	0.398
*L*	0.201	0.656	*L*	0.280	0.599
*P* × *T*	0.048	0.827	*P* × *T*	0.027	0.870
*P* × *L*	0.521	0.475	*P* × *L*	1.080	0.305
*T* × *L*	2.457	0.125	*T* × *L*	0.837	0.366
*P* × *T* × *L*	0.722	0.400	*P* × *T* × *L*	0.002	0.965
(G) Competitive effect of species *C* on *L*			(H) Competitive effect of species *L* on *C*		
*P*	3.275	*0.078*	*P*	7.435	**0.009**
*T*	0.432	0.515	*T*	3.138	*0.084*
*N*	0.147	0.703	*N*	0.869	0.357
*P* × *T*	0.019	0.892	*P* × *T*	0.026	0.872
*P* × *N*	0.713	0.404	*P* × *N*	1.229	0.274
*T* × *N*	0.038	0.845	*T* × *N*	2.646	0.112
*P* × *T* × *N*	0.300	0.587	*P* × *T* × *N*	3.867	*0.056*
(I) Competitive effect of species *P* on *T*			(J) Competitive effect of species *T* on *P*		
*C*	10.705	**0.002**	*C*	1.860	0.180
*N*	<0.001	0.988	*N*	2.989	*0.092*
*L*	1.892	0.177	*L*	2.364	0.132
*C* × *N*	1.029	0.316	*C* × *N*	0.956	0.334
*C* × *L*	0.672	0.417	*C* × *L*	0.535	0.469
*N* × *L*	0.147	0.704	*N* × *L*	1.899	0.176
*C* × *N* × *L*	0.114	0.737	*C* × *N* × *L*	0.225	0.638
(K) Competitive effect of species *P* on *N*			(L) Competitive effect of species *N* on *P*		
*C*	3.597	*0.065*	*C*	2.963	*0.093*
*T*	4.035	*0.051*	*T*	0.026	0.873
*L*	2.839	0.100	*L*	0.830	0.368
*C* × *T*	0.538	0.468	*C* × *T*	0.941	0.338
*C* × *L*	0.006	0.939	*C* × *L*	0.098	0.756
*C* × *L*	0.279	0.600	*C* × *L*	0.151	0.699
*C* × *T* × *L*	0.408	0.527	*C* × *T* × *L*	0.542	0.466
(M) Competitive effect of species *P* on *L*			(N) Competitive effect of species *L* on *P*		
*C*	7.045	**0.011**	*C*	2.624	0.113
*T*	1.720	0.197	*T*	1.240	0.272
*N*	0.109	0.743	*N*	2.818	0.101
*C* × *T*	1.884	0.177	*C* × *T*	0.307	0.582
*C* × *N*	1.248	0.271	*C* × *N*	0.190	0.665
*T* × *N*	0.004	0.951	*T* × *N*	1.522	0.224
*C* × *T* × *N*	0.046	0.832	*C* × *T* × *N*	6.193	**0.017**
(O) Competitive effect of species *T* on *N*			(P) Competitive effect of species *N* on *T*		
*C*	3.834	*0.057*	*C*	11.930	**0.001**
*P*	15.772	**<0.001**	*P*	1.780	0.190
*L*	0.805	0.375	*L*	0.000	0.990
*C* × *P*	2.288	0.138	*C* × *P*	0.856	0.360
*C* × *L*	0.876	0.355	*C* × *L*	2.497	0.122
*P* × *L*	0.509	0.480	*P* × *L*	0.253	0.618
*C* × *P* × *L*	0.208	0.651	*C* × *P* × *L*	0.353	0.556
(Q) Competitive effect of species *T* on *L*			(R) Competitive effect of species *L* on *T*		
*C*	10.624	**0.002**	*C*	4.504	**0.040**
*P*	11.070	**0.002**	*P*	6.861	**0.012**
*N*	0.082	0.776	*N*	0.448	0.507
*C* × *P*	0.467	0.499	*C* × *P*	0.013	0.910
*C* × *N*	0.031	0.861	*C* × *N*	1.985	0.167
*P* × *N*	0.021	0.886	*P* × *N*	0.045	0.834
*C* × *P* × *N*	0.196	0.660	*C* × *P* × *N*	0.494	0.486
(S) Competitive effect of species *N* on *L*			(T) Competitive effect of species *L* on *N*		
*C*	2.181	0.148	*C*	1.777	0.190
*P*	3.715	*0.061*	*P*	9.278	**0.004**
*T*	0.411	0.525	*T*	0.718	0.402
*C* × *P*	0.001	0.974	*C* × *P*	0.253	0.618
*C* × *T*	0.323	0.573	*C* × *T*	0.509	0.480
*P* × *T*	0.006	0.939	*P* × *T*	0.912	0.345
*C* × *P* × *T*	0.108	0.744	*C* × *P* × *T*	1.029	0.317

**Table 2:** Information in relation to the five species used in this study.

Species	Life Form	Morphological Features	Phenology	Typical Habitat
*Cynodon dactylon* (L.) Pers.	Perennial herb	Stems erect or creeping at the base; adventitious roots at nodes; erect parts 10–40 cm tall.	Flowering and fruiting: May–October	Roadsides, field margins, riverbanks, wastelands, hillside grasslands
*Plantago asiatica* L.	Biennial or perennial herb	Leaves basal in a rosette and broadly ovate or oblong; flowers in spikes.	Flowering: April–August; fruiting: June–September	Riverbanks, wetlands, field margins, roadsides, hillside grasslands
*Taraxacum mongolicum* Hand.-Mazz.	Perennial herb	Leaves lanceolate, 4–20 cm long; one to several scapes, 10–25 cm tall; flowers surrounded by a bell-shaped involucre.	Flowering: April–September; fruiting: May–October	Abandoned fields, hillside grasslands, roadsides, and riverbanks.
*Nepeta cataria* L.	Perennial herb	Leaves ovate or triangular-cordate; stems nearly quadrangular at the base, 40–150 cm tall.	Flowering: July–September; fruiting: September–October	Abandoned fields, field margins, roadsides, shrublands, hillside grasslands.
*Leonurus japonicus* Houtt.	Annual or biennial herb	Stems quadrangular with shallow grooves; covered with retrorse hairs, especially dense at nodes and along ridges; usually 30–120 cm tall.	Flowering: June–September; fruiting: September–October	Hillside grasslands, abandoned fields, field margins, roadsides.

**Table 3:** Information of the 31 treatments (species combinations). *C*, *P*, *T*, *N*, and *L* represent *Cynodon dactylon*, *Plantago asiatica*, *Taraxacum mongolicum*, *Nepeta cataria*, and *Leonurus japonicus*, respectively. For the five species, “1” stands for the presence of one seedling of the species and “-” means the absence of the species.

Trt. No.	*C*	*P*	*T*	*N*	*L*	Total Number of Seedlings per Pot
1	1	-	-	-	-	1
2	1	1	-	-	-	2
3	1	-	1	-	-	2
4	1	-	-	1	-	2
5	1	-	-	-	1	2
6	1	1	1	-	-	3
7	1	1	-	1	-	3
8	1	1	-	-	1	3
9	1	-	1	1	-	3
10	1	-	1	-	1	3
11	1	-	-	1	1	3
12	1	1	1	1	-	4
13	1	1	1	-	1	4
14	1	1	-	1	1	4
15	1	-	1	1	1	4
16	1	1	1	1	1	5
17	-	1	-	-	-	1
18	-	1	1	-	-	2
19	-	1	-	1	-	2
20	-	1	-	-	1	2
21	-	1	1	1	-	3
22	-	1	1	-	1	3
23	-	1	-	1	1	3
24	-	1	1	1	1	4
25	-	-	1	-	-	1
26	-	-	1	1	-	2
27	-	-	1	-	1	2
28	-	-	1	1	1	3
29	-	-	-	1	-	1
30	-	-	-	1	1	2
31	-	-	-	-	1	1

## Data Availability

Data will be made available on request.
